# Cordycepin Enhances Radiosensitivity in Oral Squamous Carcinoma Cells by Inducing Autophagy and Apoptosis Through Cell Cycle Arrest

**DOI:** 10.3390/ijms20215366

**Published:** 2019-10-28

**Authors:** Sheng-Yow Ho, Wun-Syuan Wu, Li-Ching Lin, Yuan-Hua Wu, Hui-Wen Chiu, Ya-Ling Yeh, Bu-Miin Huang, Ying-Jan Wang

**Affiliations:** 1 Department of Radiation Oncology, Chi Mei Medical Center, Liouying, Tainan 736, Taiwan; 2 Graduate Institute of Medical Sciences, Chang Jung Christian University, Tainan 711, Taiwan; 3 Department of Environmental and Occupational Health, College of Medicine, National Cheng Kung University, Tainan 704, Taiwan; 4 Department of Radiation Oncology, Chi Mei Medical Center, Tainan 710, Taiwan; 5 School of Medicine, Taipei Medical University, Taipei 110, Taiwan; 6 Department of Radiation Oncology, National Cheng Kung University Hospital, College of Medicine, National Cheng Kung University, Tainan 704, Taiwan; 7 Graduate Institute of Clinical Medicine, College of Medicine, Taipei Medical University, Taipei 110, Taiwan; 8 Division of Nephrology, Department of Internal Medicine, Shuang Ho Hospital, Taipei Medical University, Taipei 235, Taiwan; 9 Department of Cell Biology and Anatomy, College of Medicine, National Cheng Kung University, Tainan 701, Taiwan; 10 Department of Medical Research, China Medical University Hospital, China Medical University, Taichung 404, Taiwan

**Keywords:** oral squamous cell carcinoma, radiation, cordycepin, apoptosis, autophagy, cell cycle

## Abstract

Oral squamous cell carcinoma (OSCC) is one of the most common cancers worldwide and accounts for over 90% of malignant neoplasms of the oral cavity, with a 5-year survival rate of less than 50%. The long-term survival rate of OSCC patients has not markedly improved in recent decades due to its heterogeneous etiology and treatment outcomes. We investigated the anticancer effect of the combination of irradiation (IR) and cordycepin in the treatment of human OSCC cells in vitro. The type of cell death, especially autophagy and apoptosis, and the underlying mechanisms were examined. We found synergistic effects of cordycepin and IR on the viability of human oral cancer cells. The combination of cordycepin and IR treatment induced apoptosis, cell cycle arrest, and autophagic cell death. Furthermore, cordycepin induced S-phase arrest and prolonged G2/M arrest in the cells that received the combination treatment compared with those that received irradiation alone. Combined treatment induced the upregulation of ATG5 and p21 in an autophagy cascade-dependent manner, arrested the cell cycle in the G2/M phase, and repressed cell proliferation. Thus, we conclude that the combination of cordycepin and IR treatment could be a potential therapeutic strategy for OSCC.

## 1. Introduction

Oral squamous cell carcinoma (OSCC), a major subtype of head and neck carcinoma displaying many pathological differences from cancers found at other sites in the head and neck region, is among the top 12 most common cancers worldwide [1,2]. OSCC accounts for over 90% of malignant neoplasms of the oral cavity, with a 5-year survival rate of less than 50% [3]. Exposure to multiple carcinogenic factors, including tobacco and alcohol, the most dominant etiologic factors of OSCC, has been identified to critically contribute to this malignancy [4]. However, the molecular and cellular mechanisms underlying the pathogenesis of OSCC are poorly understood. In fact, surgical resection is practical for OSCC patients, but the long-term survival rate of OSCC patients has not markedly improved in recent decades. Thus, adding chemotherapy, radiotherapy, or both (chemoradiotherapy, CRT) as an adjuvant or definitive treatment is acceptable as a novel treatment strategy for cancer therapy [5]. Within the context of CRT, the optimal dose for OSCC irradiation (IR) is not clearly defined. To diminish the damage to normal tissue, treatment with chemical modifiers as radiosensitizers in combination with lower dose irradiation may augment overall therapeutic efficacy [5,6].

Ionizing radiation (IR) is one of the most effective tools in the clinical treatment of cancer, and plays a key role in therapy due to its ability to directly induce single and double strand breaks by damaging DNA, leading to cell death [7,8]. The induction of apoptotic cell death is a significant mechanism of tumor cells under the influence of radio/chemotherapy with a low propensity for apoptosis [9]. Increasing the sensitivity of tumor cells to the lethal effects of radiation has the potential to improve the efficacy of radiotherapy [10]. Studies have shown that the G2/M phase in the cell cycle is the most sensitive to radiation, raising the possibility that a G2/M inducer may act as a radiosensitizer in cancer therapy [11,12,13,14]. Differences in the length and magnitude of radiation-induced G2/M delay may be critical determinants of cellular radiosensitivity. It has been demonstrated that cordycepin can increase radiosensitivity in cervical cancer cells by overriding or prolonging radiation-induced G2/M arrest to promote apoptotic cell death [15].

Cordycepin (3′-deoxyadenosine) is a major bioactive component found in *Cordyceps sinensis* and has a wide range of biological effects in the regulation of steroidogenesis, inflammation, and platelet aggregation [16,17,18,19]. Cordycepin also exerts various antitumor and antimetastasis abilities by inducing cell cycle arrest and apoptosis [20,21]. It has been shown that cordycepin can induce apoptosis in human colorectal cancer cell lines (SW480 and SW620) by enhancing the protein expression of JNK, p38 kinase, and proapoptotic molecules [22]. Moreover, the induction of active caspase-3 and the cleavage of poly (ADP-ribose) polymerase protein (PARP) by cordycepin led to apoptotic cell death in human neuroblastoma SK-N-BE(2)-C and human melanoma SK-Mel-2 cell lines [23]. To date, there are only a few studies on cordycepin in oral cancer therapy. However, it is currently known that cordycepin can inhibits OSCC mainly by inducing apoptosis. Lin et al. reported that water extract from the mycelia of surface liquid-cultured cordyceps militaris (WECM) suppressed cell viability of SCC-4 oral cancer cells via inducing oxidative stress, mitochondrial dysfunction and cell cycle arrest at the G2/M phase [24]. Su’s research group revealed cordycepin against OSCC by apoptosis induction and epithelial-mesenchymal transition (EMT) inhibition without affecting the human fibroblasts (HFW). The group also mentioned that cordycepin may be a potential radiosensitizer [25]. Our study combined the outstanding findings of Su et al. and further validated the role of autophagy and detailed mechanism in the combination of cordycepin and radiotherapy.

G2/M arrest and apoptosis are common phenomena occurring after irradiation treatment related to DNA damage [13,26]. A previous study has demonstrated that cordycepin could arrest cell cycle at certain checkpoints related to apoptosis [20]. It is well known that key transitions in cell cycle are regulated by cyclin and cyclin-dependent kinase (CDK) molecules [27]. Studies have demonstrated that cordycepin could decrease the percentage of G1 phase cells and increase the percentage of G2/M and sub-G1 phase cells in OEC-MI human oral squamous cancer cells [20]. Another study reported that cordycepin inhibited cyclin B/CDC-complex expression and upregulated p21WAF1 expression to induce cell cycle G2/M arrest in human bladder carcinoma cells and colon cancer cells [28]. In human immortalized epithelial endometriotic cells and/or breast cancer cells, cordycepin would upregulate p21 and downregulate cyclin D1 with the reduced phosphorylation of p38 MAPK and/or retinoblastoma protein (pRb) [28,29]. However, the role of autophagy induced by the combination of cordycepin and IR treatment in oral cancer has not been fully determined.

The ubiquitin-proteasome system (UPS) and autophagy are the two major intracellular protein degradation pathways in eukaryotic cells which are responsible for degrading and recycling long-lived proteins and damaged organelles [30]. In addition to the role in cell survival, a function for autophagy in cell death has long been well proposed [31]. Studies have shown that inhibition of proteasomal activity or treatment with IR could induce autophagy, but not apoptosis, in cancer cells [32,33,34,35]. It has also been reported that an excess of autophagy induces cell death and may act as a tumor-suppressing mechanism [36].

Apoptosis, also referred to as programmed cell death, is mainly characterized by a series of distinct changes in cell morphology, such as DNA fragmentation and other biochemical changes [37]. A previous study demonstrated that cordycepin could induce apoptosis by regulating the expression of various proteins, such as the Bcl-2 family of proteins, which includes anti- and proapoptotic members, in human colorectal cancer cells [37]. During the course of apoptosis, the cleavage of caspases (cysteine-dependent aspartate specific protease), such as caspase-3 and caspase-7, is observed, which further cleaves PARP (responsible for DNA repair) [38] and results in the execution of cell death [39,40]. 

In the present study, we investigated the anticancer effect of the combination of IR and cordycepin in the treatment of in vitro human OSCC cells. The type of cell death, especially autophagy and apoptosis, and the underlying mechanisms were examined.

## 2. Results

### 2.1. Cell Death Dosage Effects of Cordycepin and/or IR on SCC-9, SCC-25, and SAS Cells

**Figure 1:**
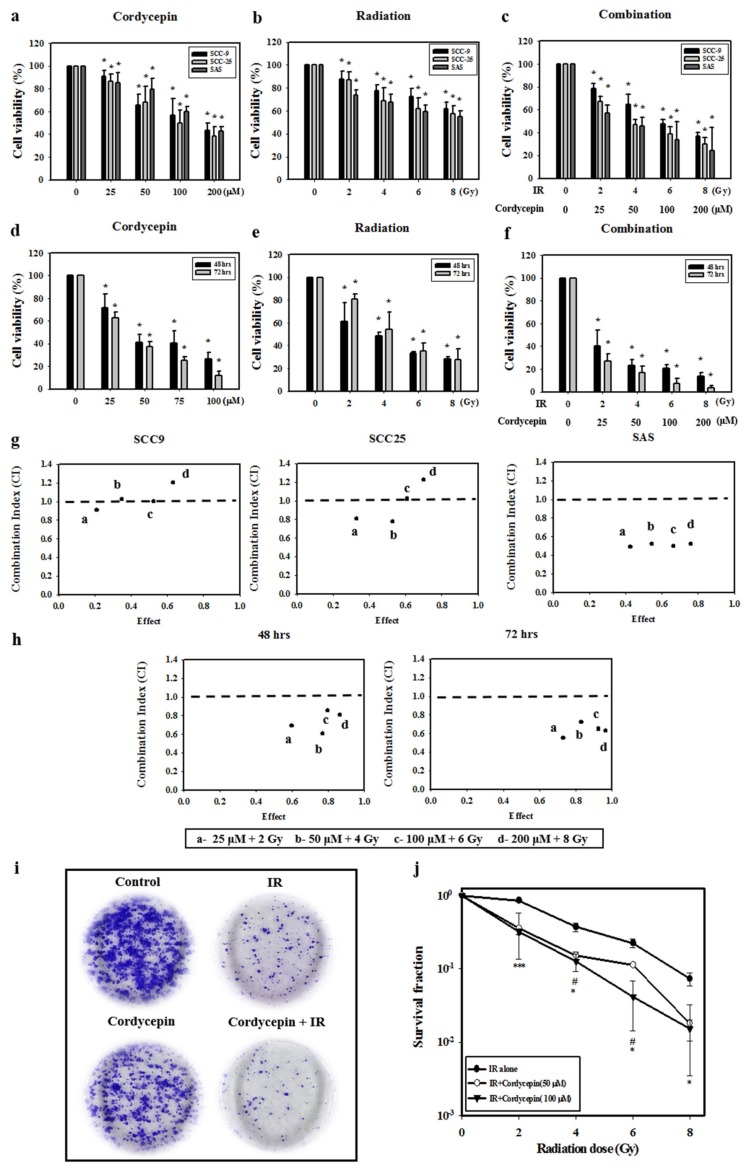
Synergistic effects of cordycepin and irradiation (IR) on the viability of human oral cancer cells. (**a**) Dose-dependent effects of cordycepin and/or IR on the viability of squamous cell carcinoma (SCC)-9, SCC-25, and SAS cells. Cells were treated with 0, 25, 50, 100, or 200 μM cordycepin for 24 h. * *p* < 0.05, control versus IR + cordycepin. (**b**) Cells were treated with 0, 2, 4, 6, or 8 Gy IR for 24 h. * *p* < 0.05, control versus IR + cordycepin. (**c**) Dose-dependent effects of cordycepin combined with IR on cell viability in SCC-9, SCC-25, and SAS cells for 24 h. * *p* < 0.05, control versus IR + cordycepin. (**d**) SAS cells were treated with 0, 25, 50, 100, or 200 μM cordycepin for 48 and 72 h. * *p* < 0.05, control versus IR + cordycepin. (**e**) SAS cells were treated with 0, 2, 4, 6, or 8 Gy IR for 48 and 72 h. * *p* < 0.05, control versus IR + cordycepin. (**f**) Dose-dependent effects of cordycepin combined with IR on cell viability in SAS cells for 48 and 72 h. (**g**) Combination index (CI) plot of cordycepin, IR, or their combination following treatment of SCC-9, SCC-25, and SAS cells for 24 h. (**h**) CI plot of cordycepin, IR, or their combination following treatment of SAS cells for 48 and 72 h. * *p* < 0.05, control versus IR + cordycepin. (**i**) Cordycepin decreases clonogenic survival in SAS cells after IR (4 Gy) treatment. Cells were plated in 6 cm dishes for 10 days. Dishes were stained with crystal violet. (**j**) Colonies containing >50 cells were scored as positive. Data presented as mean ± standard deviation from three independent experiments. ^#^ *p* < 0.05, IR alone versus IR + cordycepin (50 μM), * *p* < 0.05; ** *p* < 0.01; and *** *p* < 0.001, IR alone versus IR + cordycepin (100 μM).

Oral cancer SCC-9, SAS, and SCC-25 cells were treated with cordycepin alone (0, 25, 50, 100, or 200 μM) (Figure 1a), irradiation alone (0, 2, 4, 6, or 8 Gy) (Figure 1b), or their combination (control, 25 μM + 2 Gy, 50 μM + 4 Gy, 100 μM + 6 Gy, and 200 μM + 8 Gy) (Figure 1c) for 24 h, and cell viability was examined. The results show that cell viability among the three cell lines significantly decreased by increasing the dosage of cordycepin alone or IR alone (*p* < 0.05) (Figure 1a,b). In addition, cordycepin combined with IR treatment significantly inhibited cell viability in SCC-9, SCC-25, and SAS cells (Figure 1c) (*p* < 0.05). In fact, the combination treatment (25 μM + 2 Gy, 50 μM + 4 Gy, 100 μM + 6 Gy, and 200 μM + 8 Gy) reduced cell viability to 78.8%, 65%, 47.6%, and 36.8% in SCC-9 cells, by 57.2%, 45.5%, 33.7%, and 24.1% in SAS cells, and 67%, 47%, 39%, and 29.9% in SCC-25 cells.

The temporal effect of cordycepin and/or radiation treatment on SAS cell viability was then investigated. The results show that increasing the doses of IR (2, 4, 6, and 8 Gy) and cordycepin (25, 50, 100, and 200 μM) for 48 and 72 h significantly reduced SAS cell viability (Figure 1d,e) (*p* < 0.05). Moreover, the combined treatment of cordycepin and IR (25 μM + 2 Gy; 50 μM + 4 Gy; 100 μM + 6 Gy; and 200 μM + 8 Gy) reduced cell viability to 40.0%, 23.2%, 20.4%, and 13.5% at 48 h and 26.8%, 16.8%, 7.4%, and 3.6% at 72 h (Figure 1f).

To further determine whether the combination treatment of cordycepin and IR had a synergistic effect, the CaluSyn2.0 program was used, and the results show that the combination index (CI) values were 0.966, 1.033, 0.965, and 1.15 in SCC-9 cells, 0.492, 0.522, 0.499, and 0.521 in SAS cells, and 0.804, 0.773, 1.019, and 1.225 in SCC-25 cells (Figure 1g). Only SAS cells had CI values less than 1, indicating that all combination treatments have a synergistic effect on reducing cell viability. Thus, only SAS cells were selected for further investigation in the present study. The CI was also analyzed after treatment for 48 and 72 h, and the data showed that the CI values were all below 1 in SAS cells, indicating that the synergistic effect of combined IR and cordycepin did occur in SAS cells (Figure 1h). To further validate whether cordycepin affected radiation sensitivity, radiation dose-response survival curves were determined by a clonogenic assay (Figure 1i,j). The combined treatment of IR and cordycepin resulted in decreased survival fractions compared to cells treated with IR alone. Thus, these data highly suggest that cordycepin could enhance radiosensitivity in oral cancer cells, especially SAS cells. In addition, suboptimal combined doses of IR and cordycepin (irradiation at 4 Gy and cordycepin at 50 μM) were used to induce cytotoxicity in SAS cells to reveal the possible mechanisms involved.

### 2.2. Apoptotic Effect of Combined IR and Cordycepin on SAS Cells

To determine how the combined IR and cordycepin treatment could induce SAS cell death, a flow cytometry assay with annexin V single staining or annexin V plus propidium iodide (PI) double staining was used to determine whether the apoptotic pathway was involved. SAS cells were treated with radiation plus cordycepin (2 Gy + 25 μM, 4 Gy + 25 μM and 4 Gy + 50 μM) for 24, 48, and 72 h. The results show that the combined treatment of 2 Gy radiation and 25 μM cordycepin for 72 h significantly induced early apoptosis (21.1%) compared to radiation (13%) or cordycepin (12.3%) alone (Figure S1a) (*p* < 0.05). In addition, the combined treatment of 4 Gy radiation and 25 μM cordycepin for 48 and 72 h significantly induced early apoptosis (17.6% and 26.4%, respectively) compared to radiation alone (14% and 12.6%, respectively) and cordycepin alone (8.9% and 12.3%, respectively) (Figure S1b) (*p* < 0.05). Moreover, the combined treatment of 4 Gy radiation and 50 μM cordycepin for 48 and 72 h significantly induced early apoptosis (25% and 50%, respectively) compared to radiation alone (15% and 28%, respectively) or cordycepin alone (16% and 20%, respectively) (Figure S1c) (*p* < 0.05). These results illustrate that all combined treatments for 48 and/or 72 h could induce early apoptosis in SAS cells, and the combined treatment of 4 Gy radiation and 50 μM cordycepin for 72 h could induce approximately 50% early apoptosis in SAS cells.

**Figure 2:**
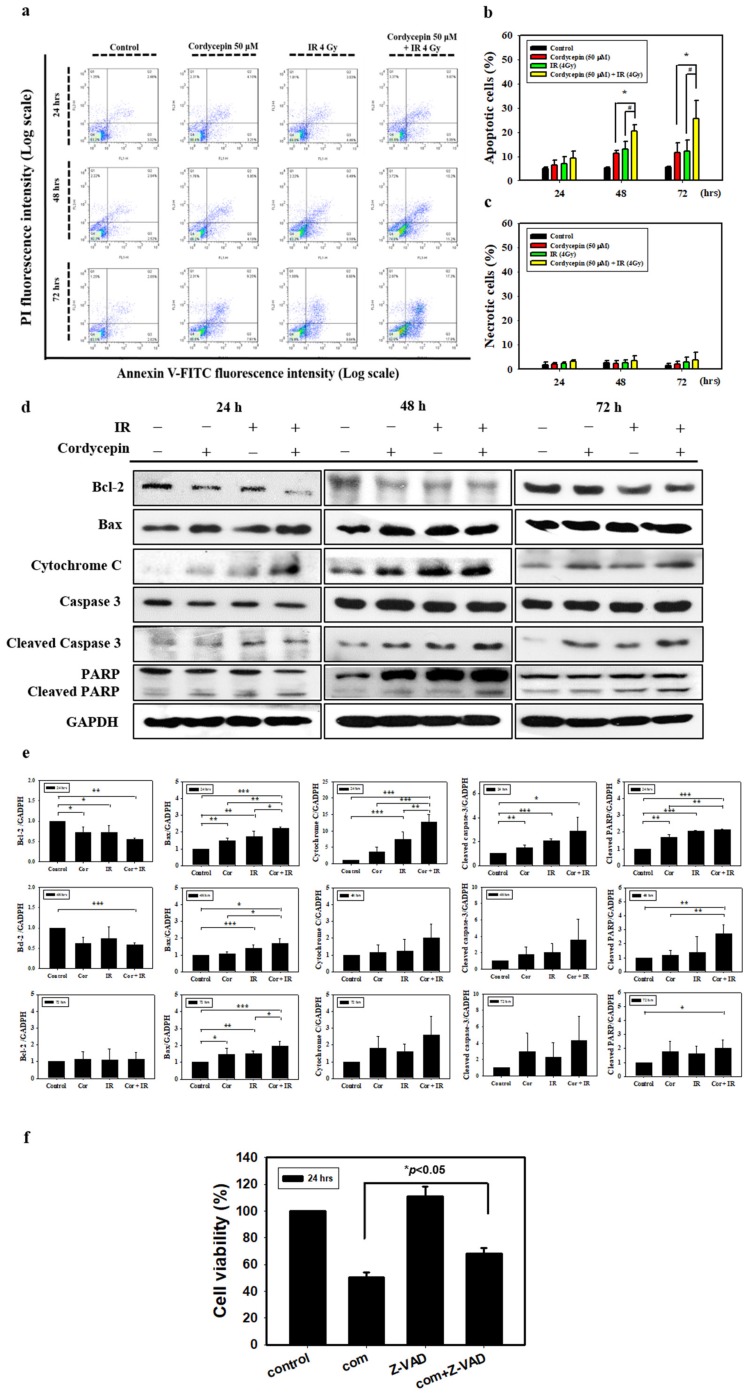
Measurement of apoptosis in SAS cells receiving various treatments. (**a**) SAS cells were treated with cordycepin and/or IR for the indicated times and stained with annexin V and propidium iodide (PI). Flow cytometry was used to determine the fractions of viable cells (annexin V−, PI−; lower left quadrant), necrotic cells (annexin V−, PI+; upper left), early apoptotic cells (annexin V+, PI−; lower right), and late apoptotic cells (annexin V+, PI+; upper right). (**b**) Quantification of apoptotic cells from flow cytometric analysis at 24, 48, and 72 h. (**c**) Quantification of necrotic cells from flow cytometric analysis at 24, 48, and 72 h. *p < 0.05, cordycepin versus IR + cordycepin, ^#^ *p* < 0.05, IR versus IR + cordycepin. (**d**) The cells were treated with 4 Gy IR and/or 50 μM cordycepin for 24, 48, and 72 h. After treatment, Bcl-2, Bax, cytochrome C, cleaved caspase3, and cleaved poly (ADP-ribose) polymerase protein (PARP) proteins were detected by Western blot. (**e**) The immunoblot represents the observations from one single experiment repeated at least three times. The integrated optical density of the protein was analyzed after normalization with glyceraldehyde 3-phosphate dehydrogenase (GAPDH) in each lane. Each datum point represents the mean ± standard error of the mean of three separate experiments. (**f**) Cells were pretreated with 10 μM pan-caspase inhibitor (Z-VAD-FMK) for 2 h and then treated with cordycepin (50 μM) and/or IR (4 Gy) for 24 h. * *p* < 0.05, IR + cordycepin versus IR + cordycepin + Z-VAD-FMK. FITC = fluorescein isothiocyanate.

To further confirm that radiation and cordycepin induced cell apoptosis, a flow cytometry assay with annexin V plus PI double staining was exploited following the combined treatment of 4 Gy radiation and 50 μM cordycepin for 24, 48, and 72 h in SAS cells. It is well established that double-negative cells are viable, annexin V single-positive cells are early apoptotic, PI single-positive cells are necrotic, and double-positive cells are late apoptotic, which can be illustrated in four quadrants [41]. The results showed that control, 50 μM cordycepin, 4 Gy radiation, and 4 Gy radiation plus 50 μM cordycepin for 48 h induced 5.4 ± 0.2, 11.4 ± 1.0, 13.0 ± 3.3 and 20.5 ± 2.6 apoptosis (early plus late apoptosis) and treatment for 72 h induced 5.4 ± 0.3, 11.6 ± 4.1, 12.0 ± 4.8 and 25.5 ± 7.7 apoptosis (Figure 2a,b). These data illustrate that 4 Gy radiation plus 50 μM cordycepin for 48 and 72 h significantly stimulated apoptosis in SAS cells (*p* < 0.05). Conversely, there were no differences in necrosis following treatment for 48 and 72 h (Figure 2a,c) (*p* > 0.05).

Since the activation of caspases plays a critical role in apoptosis, the activation of caspases in SAS cells treated with 50 μM cordycepin and/or 4 Gy radiation for 24, 48, and 72 h was investigated. Caspase-3 activity was found to increase significantly in SAS cells. The results also show that the combination treatment induced the expression of Bax and cytochrome c and the cleavage of PARP. The levels of the antiapoptotic protein Bcl-2 were decreased in SAS cells following combination treatment (Figure 2d,e). To identify the relevance of caspase activation in the combined treatment upon apoptosis, SAS cells were cultured in the presence and absence of the broad-spectrum caspase inhibitor Z-VAD-FMK and cell viability was analyzed using a trypan blue assay. As shown in Figure 2f, pretreatment of cells with Z-VAD-FMK significantly restored cell viability (*p* < 0.05). These results indicate that cordycepin and/or radiation activate caspase pathways to induce apoptotic cell death in SAS cells.

### 2.3. Autophagic Effect of Combined IR and Cordycepin on SAS Cells

**Figure 3:**
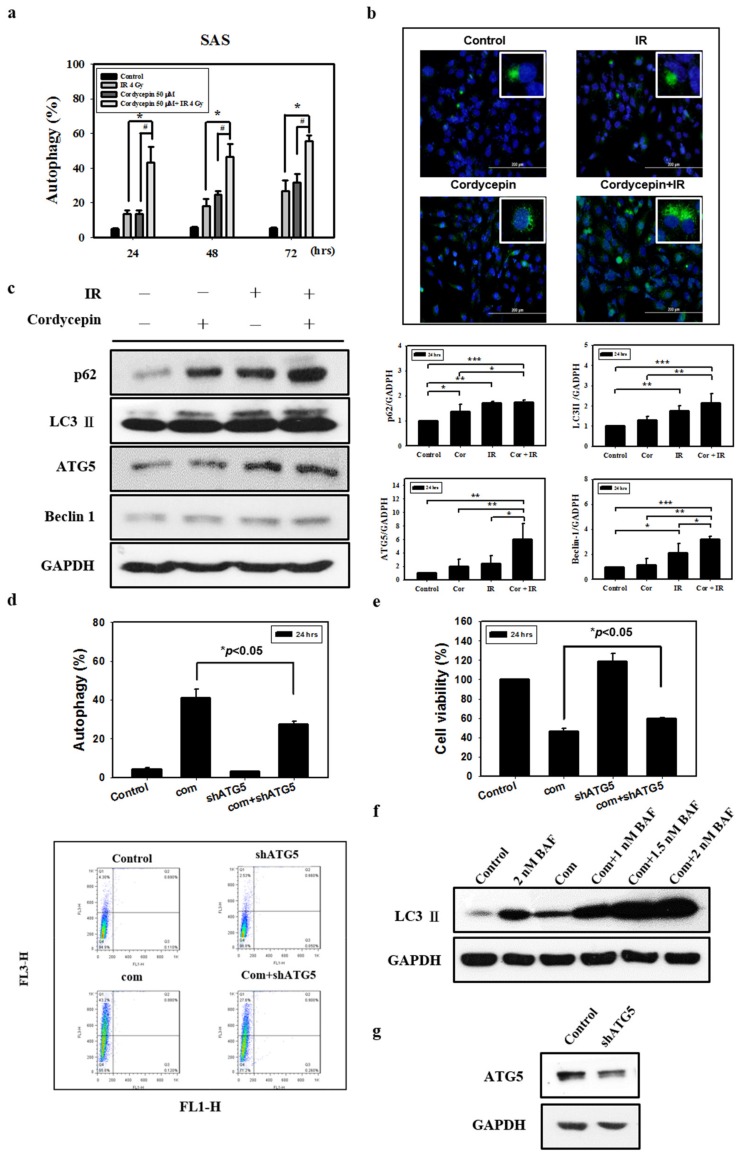
Measurement of autophagy in SAS cells receiving various treatments. Detection of green and red fluorescence in acridine orange-stained cells using flow cytometry. Quantification of acidic vesicular organelles (AVOs) with acridine orange using flow cytometry. (**a**) SAS cells were treated with IR (4 Gy) and cordycepin (50 μM) alone or their combination for 24, 48, and 72 h. ^#^ *p* < 0.05, cordycepin versus IR + cordycepin. * *p* < 0.05, IR versus IR + cordycepin. (**b**) Confocal immunofluorescence microscopy of microtubule-associated protein light chain 3 (LC3) following 24-h treatment with 4 Gy IR and 50 μM cordycepin alone or their combination. (**c**) Western blot analysis of autophagy-related protein expression in SAS cells. Effects of cordycepin and/or IR on the expression of p62, LC3-II, ATG5, and Beclin-1 protein in SAS cells. The cells were treated with IR (4 Gy) and cordycepin (50 μM) alone or their combination for 24 h. After treatment, the p62, LC3-II, ATG5, and Beclin-1 proteins were detected by Western blot. The immunoblot represents the observations from one single experiment repeated at least three times. The integrated optical density of the protein was analyzed after normalization with GADPH in each lane. Each datum point represents the mean ± standard error of the mean of three separate experiments. * *p* < 0.05; ** *p* < 0.01; and *** *p* < 0.001. (**d**) Measurement by flow cytometry with AVOs in the absence and presence of shATG5. shATG5 cells received the combined treatment (4 Gy IR and 50 μM cordycepin) for 24 h. (**e**) Cytotoxic effects in the absence and presence of shATG5 for 24 h. (**f**) Western blot analysis of LC3 expression in the absence and presence of bafilomycin A1 (BAF). Cells were pretreated with BAF for 1 h before receiving the combined treatment (4 Gy IR and 50 μM cordycepin) for 24 h. (**g**) SAS cells transfected with ATG5 shRNA.

There is no study illustrating whether the combination of radiation and cordycepin would induce autophagy and subsequent cell death in SAS cells. In fact, our abovementioned results show that 4 Gy radiation plus 50 μM cordycepin for 48 and 72 h induced 25% cell death through apoptosis. Thus, it is highly possible that the combination of radiation and cordycepin would stimulate autophagy in SAS cells. Thus, acridine orange staining with flow cytometry was used to analyze the autophagic phenomenon. The results show that 2 Gy radiation plus 25 μM cordycepin for 24, 48, and 72 h could significantly induce autophagy (17.5%, 25.7%, and 34.1%, respectively), which was higher than cells treated with cordycepin alone (9.9%, 18.3%, and 13.8%, respectively) and radiation alone (9.3%, 11.7%, and 12.9%, respectively) (Figure S2a) (*p* < 0.05). In addition, 4 Gy radiation plus 25 μM cordycepin for 24, 48, and 72 h could significantly induce autophagy (24.8%, 39.5%, and 47.5%, respectively), which was higher than in cells treated with cordycepin alone (10.2%, 18.3%, and 13.8%, respectively) and radiation alone (13.6%, 18.3%, and 26.9%, respectively) (Figure S2b) (*p* < 0.05). Moreover, 4 Gy radiation plus 50 μM cordycepin for 24, 48, and 72 h could significantly induce autophagy (43.3%, 46.4%, and 55.4%, respectively), which was higher than in cells treated with cordycepin alone (13.6%, 24.6%, and 31.8%, respectively) and radiation alone (13.5%, 18.3%, and 26.9%, respectively) treatments, respectively (Figure 3a) (*p* < 0.05).

Microtubule-associated protein light chain 3 (LC3) is widely used to monitor autophagy [42]. Thus, we applied fluorescence microscopy to determine the percentage of cells with punctate LC3 staining (Figure 3b). The results showed a significant increase in LC3 immunopositive dots in SAS cells that received the combined treatment compared with those in cells treated with either cordycepin alone or IR alone.

Many studies have shown that the expression of LC3 is stimulated as autophagy occurs among various cell types, and cytoplasmic LC3-I is converted to LC3-II existing on autophagosomes [43]. To confirm that the combination of radiation and cordycepin induces autophagy in SAS cells, the expression of LC3-II and related proteins (p62, ATG5, and Beclin 1) was determined by Western blotting. The data showed that the expression of p62, LC3-II, ATG5 and Beclin 1 was significantly stimulated by IR (4 Gy) and cordycepin (50 μM) for 24 h compared to the control cells and cells that received either IR alone or cordycepin alone (Figure 3c) (*p* < 0.05).

To generate an ATG5-knockdown stable cell line, SAS cells were transfected with a lentiviral vector containing short hairpin RNA (shRNA) (Figure 3g) that significantly suppressed the induction of acidic vesicular organelles (AVOs) in SAS cells after combined treatment (Figure 3d) (*p* < 0.05). In addition, we examined whether shATG5 could rescue cell viability upon the combined treatment, and the results show that compared to the combined treatment group, the ATG5-knockdown stable cell line with combined treatment displayed a significant increase in cell viability (Figure 3e) (*p* < 0.05).

We further confirmed the pro-death role of autophagy in combined treatment-induced cytotoxicity by inhibiting autophagy flux with bafilomycin A1 (BAF), an inhibitor of autophagosome-lysosome fusion [44]. BAF augmented combined treatment-induced LC3-II accumulation, indicating that autophagy flux was prevented (Figure 3f). These results show that the combination of cordycepin and IR induced autophagic cell death and profoundly suggest that autophagy induced by the combination of radiation and cordycepin could be more important than apoptosis in SAS cells.

### 2.4. Cell Cycle Effect of Combined IR and Cordycepin on SAS Cells

**Figure 4:**
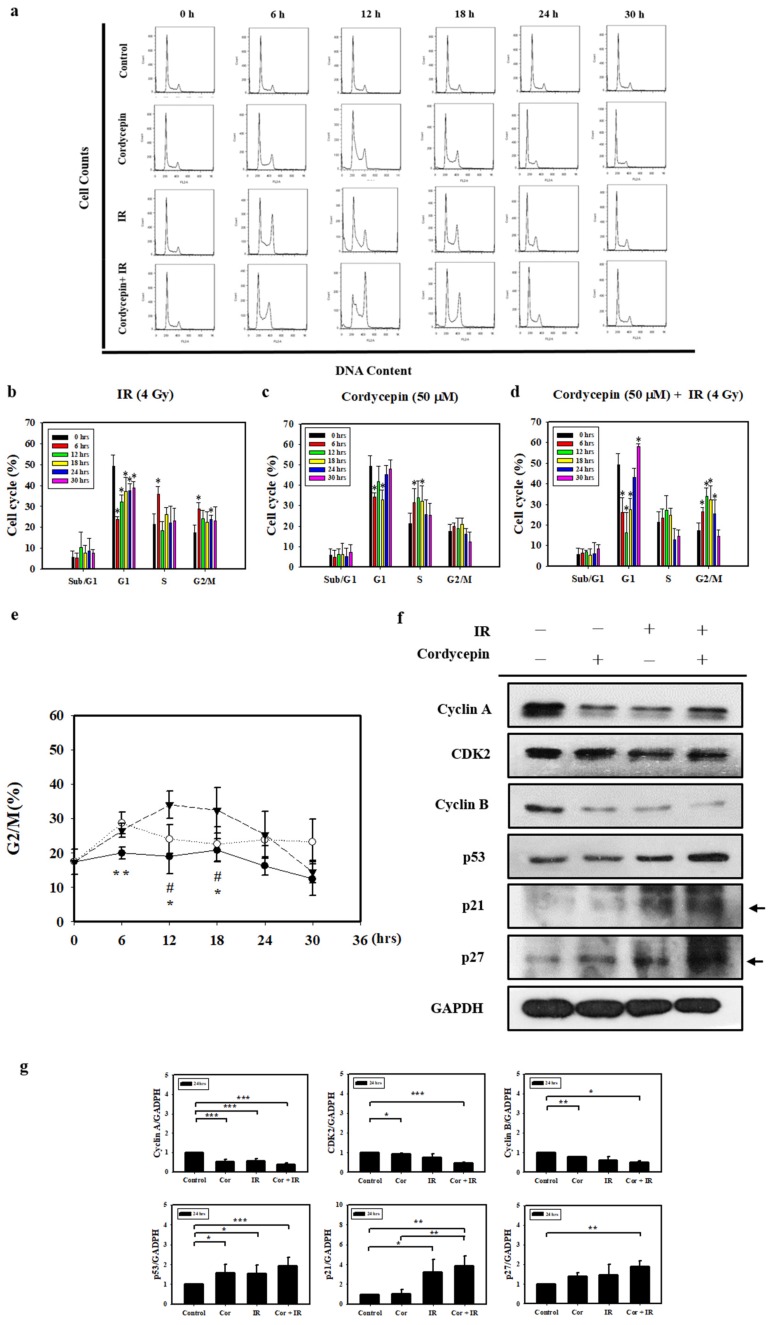
Cell cycle distribution of SAS cells treated with cordycepin and/or IR. (**a**) Time-dependent effects of cordycepin and/or IR on cell cycle distribution in SAS cells. Cells were treated with 50 μM cordycepin and/or 4 Gy IR for 6, 12, 18, 24, and 30 h. (**b**) The effects of IR on the cell cycle in SAS cells. Cells were treated with 50 μM cordycepin for 6, 12, 18, 24, and 30 h. * *p* < 0.05, treated groups versus 0 h control. (**c**) The effects of cordycepin on the cell cycle in SAS cells. Cells were treated with 50 μM cordycepin for 6, 12, 18, 24, and 30 h. * *p* < 0.05, treated groups versus 0 h control. (**d**) The effects of cordycepin and IR treatment alone or their combination on the cell cycle in SAS cells. Cells were treated with 50 μM cordycepin and 4 Gy IR for 6, 12, 18, 24, and 30 h. * *p* < 0.05, treated groups versus 0 h control. (**e**) Quantification of G2/M phase. Cells were treated with cordycepin (50 μM) or IR (4 Gy) for 6, 12, 18, 24, and 30 h. ** *p* < 0.05, IR versus cordycepin. * *p* < 0.05, cordycepin + IR versus cordycepin. ^#^ *p* < 0.05, cordycepin + IR versus IR. (**f**) Expression of cell cycle regulatory proteins in IR- and cordycepin-treated SAS cells. Effects of cordycepin and/or IR on the expression of cyclin A, cyclin E, cyclin-dependent kinase (CDK)2, cyclin B, p53, p21, and p27 proteins in SAS cells. The cells were treated with 4 Gy IR and/or 50 μM cordycepin for 24 h. After treatment, the cyclin A, cyclin E, CDK2, cyclin B, p53, p21, and p27 proteins were detected by Western blot. (**g**) The immunoblot represents the observations from one single experiment repeated at least three times. The integrated optical density of the protein was analyzed after normalization with GADPH in each lane. Each datum point represents the mean ± standard error of the mean of three separate experiments. * *p* < 0.05; ** *p* < 0.01; and *** *p* < 0.001.

Since irradiation combined with cordycepin inhibited oral cancer cell growth, we further examined whether the combined treatments caused cell death by cell cycle arrest. SAS cells growing exponentially were exposed to 4 Gy irradiation alone, 50 μM cordycepin alone, or their combination, and the percentages of each cell cycle fraction were observed at different time points (0–30 h) after treatment. Figure 4a shows the time kinetic studies of the cell cycle. As shown in Figure 4b, 4 Gy irradiation increased the population of SAS cells in the G2/M fraction at 6 h and decreased the cell population at 12 and 18 h. As shown in Figure 4c, 50 μM cordycepin increased the population of SAS cells in S phase from 12 to 30 h. As shown in Figure 4d, there was a significant amount of cell accumulation at G2/M phase in SAS cells treated with 4 Gy irradiation and 50 μM cordycepin. Figure 4e shows the time kinetic studies of the G2/M arrest effects of 4 Gy irradiation alone, 50 μM cordycepin alone, and the combined treatment on SAS cells. A significant increase in G2/M arrest was found in the cells treated with cordycepin alone and the combined treatment compared to those treated with irradiation alone at 6 h, and prolonged G2/M arrest was observed in cells that received the combined treatment compared to those that received cordycepin alone or irradiation alone at 12 and 18 h.

To examine the molecular mechanisms of G2/M arrest in SAS cells treated with IR and cordycepin, we studied the expression of G2/M-related cell cycle regulatory proteins. Cyclin A and cyclin B play important roles in both the S and G2/M phase transitions [45,46]. Figure 4f,g show the decreased expression of cyclin A, CDK2, and cyclin B, confirming S phase arrest and G2/M arrest after treatment with 4 Gy irradiation and 50 μM cordycepin alone or their combination for 24 h. In addition, the expression levels of p53 in SAS cells were upregulated in response to the combined treatment (Figure 4f,g). Moreover, the results showed significant increases in p21 and p27 expression in SAS cells (Figure 4f,g). These data suggest that p53/p21 axis signaling is likely involved in G2/M arrest following the combined treatment in SAS cells.

### 2.5. Cordycepin and/or IR Induce S Phase Arrest and Regulate the Expression of Cell Cycle Regulatory Proteins in SAS Cells

**Figure 5:**
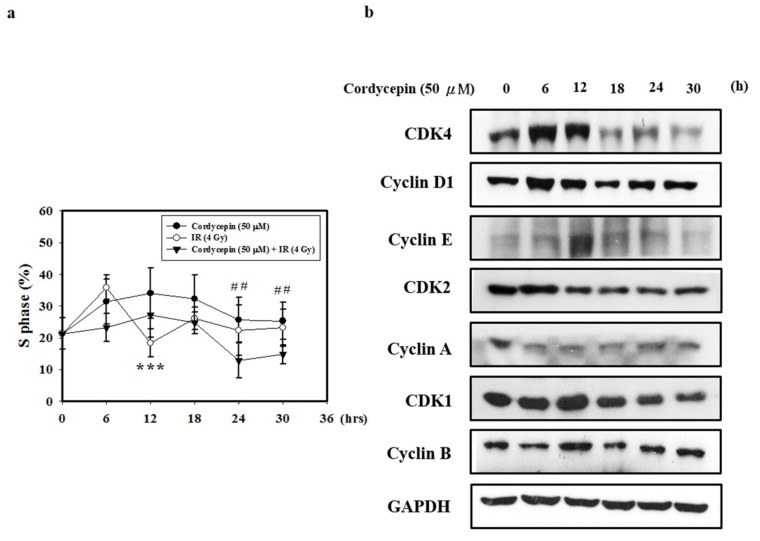
Cordycepin induces S phase arrest and regulates the expression of cell cycle regulatory proteins in IR- and cordycepin-treated SAS cells. (**a**) Quantification of S phase. Cells were treated with cordycepin (50 μM) or IR (4 Gy) for 6, 12, 18, 24, and 30 h, and DNA content was analyzed by flow cytometry. *** *p* < 0.05, cordycepin versus IR. ^##^ *p* < 0.05, cordycepin versus cordycepin + IR. (**b**) Effects of cordycepin and/or IR on the expression of CDK4, cyclin D1, CDK2, cyclin E, cyclin A, CDK1, and cyclin B proteins in SAS cells. The cells were treated with 50 μM cordycepin for 0, 6, 12, 18, 24, and 30 h. After treatment, the CDK4, cyclin D1, CDK2, cyclin E, cyclin A, CDK1 and cyclin B proteins were detected by Western blot.

Cyclin A/CDK2 are crucial for maintaining G1 to S checkpoint activation, DNA repair, and apoptosis [47]. To determine the effect of cordycepin and/or IR on the cell cycle distribution of SAS cells, we performed flow cytometry assays. As shown in Figure 5a, SAS cells were treated with cordycepin (50 μM) and/or IR (4 Gy) for 0–30 h, and the results show that cordycepin alone significantly induced S phase arrest compared with the combined treatment in SAS cells.

Cyclin A and CDK2 are two key regulators of S phase [48]; thus, we also investigated the effects of cordycepin alone on S-phase cell cycle-related proteins (Figure 5b). The results show that after treatment with cordycepin (50 μM) for 0–30 h, the expression of cell cycle regulatory proteins (CDK4, Cyclin D1, CDK2, Cyclin E, Cyclin A, CDK1, and Cyclin B proteins) decreased in a time-dependent manner. These results indicate that cordycepin could arrest the cell cycle at S phase in a time-dependent manner, which could suppress tumor growth by preventing proper DNA replication.

### 2.6. Cordycepin Combined with IR Induced G2/M Cell Cycle Arrest through Autophagy Induction in SAS Cells

**Figure 6:**
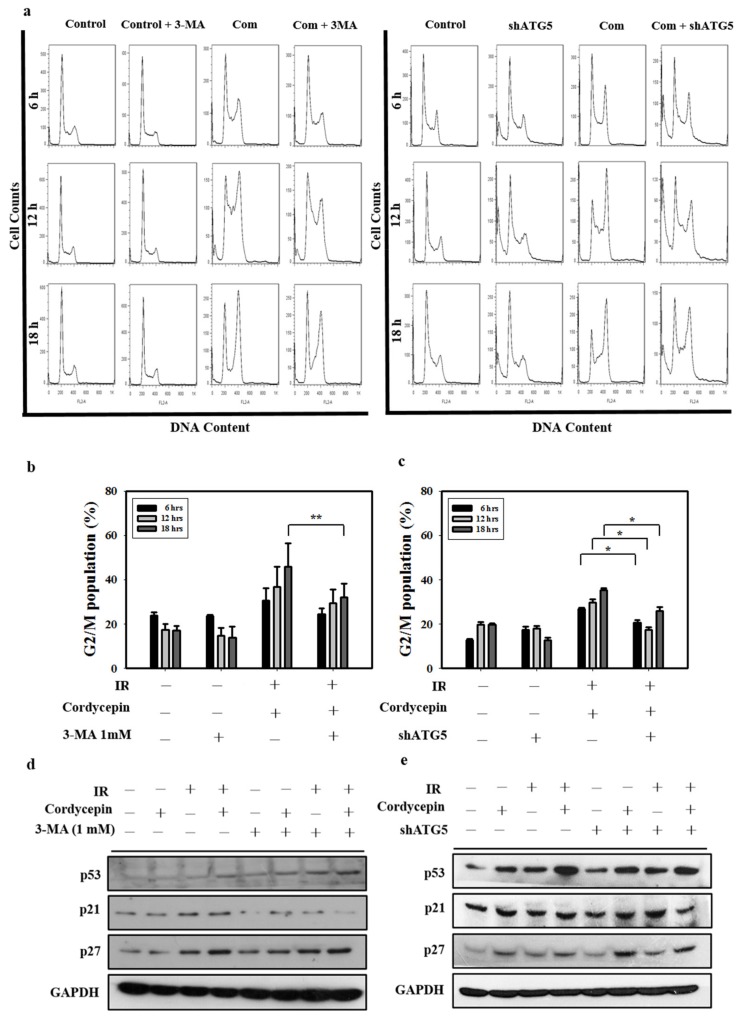
Cordycepin and IR induce G2/M cell cycle arrest through autophagy induction in oral cancer cells. (**a**) Cell cycle distribution after treatment with 3-MA (1 mM) and shATG5 plus cordycepin (50 μM) and/or IR (4 Gy) for 6, 12, and 18 h in SAS cells. (**b**) Quantification of G2/M phase. Cells were pretreated with 3-MA (1 mM) followed by cordycepin (50 μM) and/or IR (4 Gy) for 6, 12, and 18 h. ** *p* < 0.01. (**c**) Quantification of G2/M phases. SAS cells were transfected with ATG5 shRNA and treated with cordycepin (50 μM) and/or IR (4 Gy) for 6, 12, and 18 h. * *p* < 0.05. (**d**) Western blot analysis of p53, p21, and p27 expression in the absence and presence of 3-MA. Cells were pretreated with 3-MA (1 mM) for 1 h before receiving the combined treatment (4 Gy IR and 50 μM cordycepin) for 24 h. (**e**) Western blot analysis of p53, p21, p27, and ATG5 protein expression in SAS cells transfected with ATG5 shRNA. shATG5 cells were treated with 4 Gy IR and/or 50 μM cordycepin for 24 h.

To further confirm whether G2/M cell arrest and autophagy play important roles in cell death induced by cordycepin combined with IR in SAS cells, 3-MA and ATG5 shRNA were exploited in SAS cells, and the G2/M cell population was determined. Figure 6a–c shows that treatment with cordycepin (50 μM) combined with IR (4 Gy) for 6, 12, and 18 h increased the G2/M cell population compared with the control group. When the autophagy cascade was blocked by 3-MA and SAS cells were transfected with ATG5 shRNA, cordycepin (50 μM) combined with IR (4 Gy) for 6, 12, and 18 h did not increase the G2/M cell population (Figure 6a–c), suggesting that cordycepin combined with IR induced cell cycle arrest in an autophagy cascade-dependent manner.

p21 is a regulator that prevents cell cycle progression from the G2 to the M phase [49]. Previous studies have found that the expression of p21 is upregulated after treatment with some autophagy inducers [50,51]. However, whether cordycepin combined with IR could upregulate p21 expression through an autophagy cascade remains unclear. In the present study, p53, p21, and p27 protein levels increased following cordycepin combined with IR treatment, as measured by Western blot. The results show that the expression of p53, p21, and p27 was induced by cordycepin combined with IR treatment in SAS cells (Figure 6d,e). However, pretreatment with 3-MA or transfection with ATG5 shRNA attenuated the expression of p21, but not p53 and p27, in cells that received cordycepin combined with IR treatment (Figure 6d,e), suggesting that cordycepin combined with IR upregulated p21 in an autophagy cascade-dependent manner. Thus, cordycepin combined with IR treatment induced the upregulation of ATG5, triggered autophagy, upregulated p21, arrested the cell cycle in G2/M phase, and induced SAS cell death.

## 3. Discussion

OSCC accounts for over 90% of malignant neoplasms of the oral cavity, and the mortality rate has remained largely unchanged for the past decade, with a 5-year survival rate of less than 50% [3]. In fact, the classification of tumor staging criteria regarding depth of invasion, extranodal extension, and lymph node ratio to identify patients affected by squamous cell carcinoma of the tongue (SCCT) with a poor prognosis remain inconclusive [52]. Moreover, the molecular and cellular mechanisms underlying the pathogenesis of OSCC are poorly understood. Cancer therapy has increasingly focused on novel treatment strategies combining radiotherapy and chemotherapy [5,6]. In the present study, we investigated the anticancer effect of the combination of IR and cordycepin in human OSCC cells. IR is one of the most effective tools in the clinical treatment of cancer and plays a key role in therapy due to its ability to directly induce single- and double-strand breaks by damaging DNA, leading to cell death [7,8]. Cordycepin (3′-deoxyadenosine) is a major bioactive component found in *Cordyceps sinensis*. Moreover, cordycepin has been shown to exert a large variety of antitumor abilities [20,21]. In fact, studies have demonstrated that chemotherapy and radiation can induce cell death through autophagy [53]. The primary objective of the current study was to examine synergistic effects the combined treatment with different agents. We demonstrated that the combination of cordycepin and IR resulted in a synergistic cell-killing effect in SAS human oral cancer cells in vitro.

Apoptosis, also referred to as programmed cell death, is characterized mainly by a series of distinct changes in cell morphology, such as DNA fragmentation with various biochemical changes [37]. Previous research has demonstrated the role of cordycepin in the induction of apoptosis by regulating the expression of various proteins, such as the Bcl-2 family of proteins that includes anti- and proapoptotic members [37]. The induction of apoptotic cell death is a significant mechanism of tumor cells under the influence of radio/chemotherapy with a low propensity for apoptosis [9]. Increasing the sensitivity of tumor cells to the lethal effects of radiation has the potential to improve the efficacy of radiotherapy [10]. Moreover, study has shown that the expression of survivin in nucleus seems to suggest a poor prognosis in OSCC patients with more aggressive and disseminated disease influencing follow-up and therapeutic protocols [54]. In the present study, Bcl-2 protein expression was downregulated by cordycepin and IR, whereas Bax, cytochrome c, cleaved caspase-3, and cleaved PARP were upregulated in cells that received the combined treatment. Our data regarding the activation of the caspase cascade by the combination of cordycepin and IR in SAS cells are solid and comparable to other studies [9,10,38].

Previous reports have illustrated that the therapeutic effect of radiotherapy can be influenced by regulating autophagy [55]. When the level of autophagy is higher than the tolerance of cells, radiation sensitization can be enhanced, which can lead to autophagic cell death [56]. We found that cordycepin combined with IR induced a significant amount of autophagy in SAS cells. The ATG5 knockdown and 3-MA inhibitor experiments further rescued SAS cell viability, which was decreased by the combination of cordycepin and IR. In addition, our results also show that the combined treatment significantly induced the accumulation of LC3-II in the presence of BAF. Thus, the combined treatment of cordycepin with IR increased autophagic flux and induced autophagic cell death in SAS cells.

G2/M arrest and apoptosis are common phenomena after DNA damage that occurs after irradiation [13]. The rapidly dying and radiosensitive cells undergo apoptosis at different points in the cell cycle, whereas the slowly dying cells show a variety of cell cycle arrest profiles, initiating apoptosis only after an accumulation of cells in the G2/M fraction [26]. In the present study, we conducted cell cycle distribution assays and apoptosis induction using 4 Gy irradiation alone and in combination with cordycepin. The 4 Gy irradiation for 12 h induced remarkable G2/M arrest and the combined treatment significantly prolonged G2/M fraction arrest which consequently induced cell-killing effects. In addition, IR and cordycepin modulated the expression of cyclin A, CDK2, and cyclin B, resulting in the S and G2/M phase transitions, consistent with other studies [45,46].

When a cell is subject to external stimulation, the regulatory proteins p53, p21, and p27 are activated to interact with the cyclin/CDK complex, which could interrupt the conformational change and cause cell cycle arrest [57]. Previous reports have indicated that the anticancer activity of cordycepin in human bladder carcinoma cells inhibited the cyclin B/CDC complex and upregulated p21WAF1 expression to induce G2/M arrest, and similar results were observed in a study of colon cancer cells [28]. In our study, the expression levels of p53, p21, and p27 in SAS cells were also upregulated in response to cordycepin and/or the combined treatment. These data suggest that p53/p21 axis signaling is likely involved in G2/M arrest in SAS cells following the combined treatment. Our data implicate that the balance between the extent of DNA damage and the duration of G2/M arrest and S phase arrest might determine whether irradiated cells survive or undergo apoptosis.

The cell cycle is regulated by cyclin, CDKs, and CDK inhibitors [49]. Cyclin D1/CDK4 are critical kinases in cell cycle regulation that regulate normal development and tumorigenesis [58,59]. The cyclin B/CDK1 complex is the principal CDK that facilitates G2/M cell cycle transition [60,61]. Cyclin E plays a role in promoting S phase entry [62]. The cyclin A/CDK2 complex is crucial for maintaining G1 to S checkpoint activation, DNA repair, and apoptosis [47]. In the present study, cordycepin promoted the accumulation of SAS cells in S phase at 12 h. Meanwhile, cell cycle related proteins were decreased in a time-dependent manner by cordycepin in SAS cells, indicating that cordycepin induced S phase cell cycle arrest in a time-dependent manner and suppressed tumor growth by preventing proper DNA replication.

Cell cycle arrest would sufficiently induce an autophagic phenotype in cancer, which can induce cell cycle arrest [63]. Previous studies have shown that autophagy induction upregulates p21 expression [50] and that p21 overexpression can also trigger autophagy [63]. Thus, the authors of the previous studies proposed that p21 may be a mediator between autophagy and G2/M cell cycle arrest [29]. Therefore, we surveyed whether the combination of cordycepin and IR could induce G2/M cell cycle arrest through an autophagy-dependent pathway. The results show that the combined treatment promoted cell cycle arrest in G2/M phase in SAS cells. Moreover, 3-MA inhibitor and ATG5 shRNA experiments reversed autophagy phenomena following the combined treatment, suggesting that cordycepin combined with IR induced cell cycle arrest in an autophagy cascade-dependent manner. Previous studies have found that the expression of p21 was upregulated after treatment with some autophagy inducers [50,51]. In the present study, p53, p21, and p27 protein levels were increased by the combined treatment of cordycepin and IR. In addition, pretreatment with 3-MA or transfection with ATG5 shRNA attenuated the combined treatment-induced upregulation of p21 but not p53 and p27. Thus, the combined treatment of cordycepin and IR could induce the upregulation of ATG5 and trigger autophagy through the upregulation of p21 in an autophagy cascade-dependent manner that is related to cell cycle arrest in G2/M phase to induce cell death in SAS cells. In conclusion, cordycepin could enhance radiosensitivity in SAS cells by inducing autophagy and apoptosis through cell cycle arrest, which is involved in the activation of caspases, autophagy, cell cycle pathways, and p21 activation.

## 4. Materials and Methods

### 4.1. Cell Culture

Human oral cancer cell lines SCC-9 (ATCC CRL-1629) and SCC-25 (ATCC CRL-1628) were purchased from the American Type Culture Collection (ATCC), and SAS (JCRB0260) was purchased from the Japanese Collection of Research Bioresources (JCRB, Osaka, Japan). The cells were maintained in 1:1 mixture of Dulbecco’s Modified Eagle’s Medium (DMEM) and Ham’s F-12 Nutrient Mixture (Life Technologies, Paisley, UK) supplemented with antibiotics containing 100 U/mL penicillin, 100 mg/mL streptomycin (Gibco BRL, Grand Island, NY, USA) and 10% fetal bovine serum (Caisson Labs, Logan, UT, USA). All the cells were incubated in a humidified atmosphere containing 5% CO_2_ at 37 °C. Exponentially growing cells were detached using 0.05% trypsin-EDTA (Gibco BRL, Grand Island, NY, USA) in medium.

### 4.2. Irradiation Treatment, Cell Viability, and Synergistic Interaction Analysis

Irradiation was performed with 6 MV X-rays using a linear accelerator (Digital M Mevatron Accelerator, Siemens Medical Systems, Pleasanton, CA, USA) at a dose rate of 5 Gy/min. An additional 2 cm of a tissue-equivalent bolus was placed on the top of the plastic tissue-culture flasks to ensure electronic equilibrium and 10 cm of tissue-equivalent material was placed under the flasks to obtain full backscatter. After IR treatment, cells were treated with cordycepin immediately. The treated cells were centrifuged and resuspended with appropriate amount of PBS. For the cell viability assay, 20 μL cell suspension was mixed with 20 μL Trypan blue solution (0.4% in PBS). Placing the mixture on a hemocytometer, the blue-stained cells were counted as nonviable. The effect of the combination treatment was evaluated by the combination index method using CalcuSyn software (Biosoft, Paris, France), which is based on the median effect model of Chou and Talalay [64]. The experimental data were entered into the CalcuSyn interface and CI values were calculated. CI < 1, CI = 1, and CI > 1 indicate synergism, additive effect, and antagonism, respectively.

### 4.3. Clonogenic Assay

The cells were irradiated using dosages of 2, 4, 6, or 8 Gy. Cordycepin was added to the cells at concentrations of 50 or 100 μM. Cells were trypsinized and counted. Known numbers of cells were subsequently replated in 6-cm culture dishes and returned to the incubator to allow for colony development. After two weeks, colonies (containing ≥50 cells) were stained with 0.5% crystal violet solution. The plating efficiency (PE) is the ratio of the number of colonies to the number of cells seeded in the non-irradiated group. Calculation of survival fractions (SFs) was performed using the equation:

(1) $$\mathrm{SF}=\frac{\mathrm{colonies}\;\mathrm{counted}}{\mathrm{cells}\;\mathrm{seeded}\times \mathrm{PE}}$$

### 4.4. Early Apoptosis and Autophagy Detections

Apoptosis was assessed by observing the translocation of phosphotidyl serine to the cell surface, as detected with an annexin V apoptosis detection kit (Calbiochem, San Diego, CA, USA), according to our previous report [65,66]. For the autophagy analysis, cell staining with acridine orange (Sigma Chemical Co., St. Louis, MO, USA) was performed according to published procedures [67,68], adding a final concentration of 1 µg/mL for a period of 20 min. Flow cytometry was used to detect annexin V-positive cells and AVOs.

### 4.5. Stable Knockdown Clone Selection

For generation of an ATG5-knockdown stable cell line, SAS cells were transfected with lentiviral vector containing shRNA purchased from the National RNAi Core Facility located at the Institute of Molecular Biology/Genomic Research Center, Academia Sinica, Taipei, Taiwan. The clone was identified as TRCN0000151963, which targeted the human ATG5 transcript sequences, 5′-CCTGAACAGAATCATCCTTAA-3′. We added the lentivirus to cells in a growth media containing 8 μg/mL polybrene (MOI = 3). After 48 h post infection, we removed the media and replaced it with media containing puromycin (0.4 μg/mL) and then amplified the cells.

### 4.6. Annexin V/Propidium Iodide (PI) Double Staining Assay

After harvesting cells by trypsin and washing it by 2 mL culture medium, cell suspensions were centrifuged at 300× *g* for 10 min at 4 °C. The pellets were resuspended with cold isoton II and centrifuged again. The pellets were mixed with 100 μL staining solution for 15 min according to the user’s manual of annexin V-FITC (fluorescein isothiocyanate) apoptosis detection kit from Strong Biotech (Taipei, Taiwan). The stained cells were analyzed at λ = 488 nm excitation using 515 nm band pass filter for FITC detection and >600 nm band pass filter for PI detection by FACScan flow cytometer (Becton-Dickinson, Mountain View, CA, USA). The double-negative cells (viable), annexin V single-positive cells (early apoptotic), PI single-positive cells (necrotic), and double-positive cells (late apoptotic) could be illustrated in four quadrants [69].

### 4.7. Immunofluorescence and Confocal Microscopy

The cells were cultured on coverslips. After treatment, cells were fixed in 4% paraformaldehyde and blocked with 1% BSA for 30 min. This was followed by incubation with a specific antibody against LC3 (Cell Signaling Technology, Danvers, MA, USA) for 1 h. After washing, the cells were labeled with a DyLight™ 488-conjugated affinipure goat anti-rabbit IgG (Jackson Immuno-Research Laboratories, West Grove, PA, USA) for 1 h. Finally, the cells were stained and washed, sealed by mounting a coverslip with an appropriate mountant stabilizer, and examined with a confocal microscope (Carl Zeiess LSM780, Instrument Development Center, NCKU, Tainan, Taiwan).

### 4.8. Western Blotting

SAS cells were lysed, separated by 6–15% SDS-PAGE, transferred to a nitrocellulose membrane and then blocked with skimmed milk. GAPDH expression served as the protein loading control. The following antibodies were used for immunoblotting (IB): anti-GAPDH, cyclin B, cyclin D1, CDK2, CDK4, and CDK1 were obtained from Abcam (Cambridge, MA, USA); anti-LC3 and ATG5 were obtained from Abgent (San Diego, CA, USA); anti-p53 and p27 were obtained from GeneTex (Irvine, CA, USA); anti-p62/SQSTM1 was obtained from MBL (Nagoya, Japan); anti-PARP, cytochrome C, Bax, Beclin 1, and cyclin E were obtained from Cell Signaling Technology (Ipswich, MA, USA); anti-phospho-IkBα (S32/S36) was obtained from R&D Systems (Minneapolis, MN, USA); anti-active caspase 3 was obtained from BioVision (San Francisco, CA, USA); anti- Bcl-2 and cyclin A were obtained from Santa Cruz Biotechnology (Santa Cruz, CA, USA); and anti-p21 was obtained from Thermo (Waltham, MA, USA). Z-VAD-FMK and 3-methyladenine (3-MA) was obtained from Sigma-Aldrich (St. Louis, MO, USA). After mixing with 4× sample dye, the samples were heated at 95 °C for 5 min, placed on ice for 5 min, and subjected to a Western blot analysis.

### 4.9. Cell Cycle Analysis

A total of 1 × 10^6^ human SAS cells were suspended in ice-cold PBS and fixed in 70% ethanol at 4 °C for at least 24 h. After fixation, cells were washed twice, incubated in 0.5 mL of 0.5% Triton X-100/PBS at 37 °C for 30 min with 1 μg/mL of RNase A and stained with 0.5 mL of 50 μg/mL PI. Fluorescence emitting from the PI-DNA complex was analyzed at 488 nm/600 nm (excitation/emission wavelength) by a fluorescence-activated cell sorter (FACScan flow cytometry, Becton-Dickinson, Mountain View, CA, USA). The population of nuclei in each phase of the cell cycle was determined using Cell Quest and analyzed by WinMDI software programs (Scripps, La Jolla, CA, USA).

### 4.10. Statistical Analysis

We evaluated the differences in the differences in continuous variables (presented as mean ± standard deviation [SD]) between the groups using the two-sample t-test or one-way analysis of variance followed by a post-hoc Bonferroni test. We performed all statistical analyses using the SPSS 17.0 statistical software (SPSS Inc., Chicago, IL, USA). All statistical tests were performed at a two-sided significance level of 0.05.

## 5. Conclusions

In conclusion, cordycepin could enhance radiosensitivity in SAS cells by inducing autophagy and apoptosis through cell cycle arrest, which is involved in the activation of caspases, autophagy, cell cycle pathways, and p21 activation.

## Supplementary Materials

Supplementary materials can be found at https://www.mdpi.com/1422-0067/20/21/5366/s1. Figure S1. Measurement of apoptosis in SAS cells receiving various treatments. Figure S2. Measurement of autophagy in SAS cells receiving various treatments.

## Abbreviations

ART	artesunate
AVO	acidic vesicular organelle
BAF	bafilomycin A1
CDK	cyclin-dependent kinase
CI	combination index
EMT	epithelial-mesenchymal transition
IR	irradiation
IR	ionizing radiation
OSCC	oral squamous cell carcinoma
PARP	poly (ADP-ribose) polymerase
PE	plating efficiency
pRB	retinoblastoma protein
SF	survival fraction
shRNA	short hairpin RNA
UPS	ubiquitin-proteasome system
