# Supplementary material for: Cordycepin Enhances Radiosensitivity in Oral Squamous Carcinoma Cells by Inducing Autophagy and Apoptosis Through Cell Cycle Arrest
Source: Int J Mol Sci. 2019 Oct 28;20(21):5366. doi: 10.3390/ijms20215366 (PMC6862293; doi:10.3390/ijms20215366)
Supplement: Supplementary file 1 [file ijms-20-05366-s001.pdf]

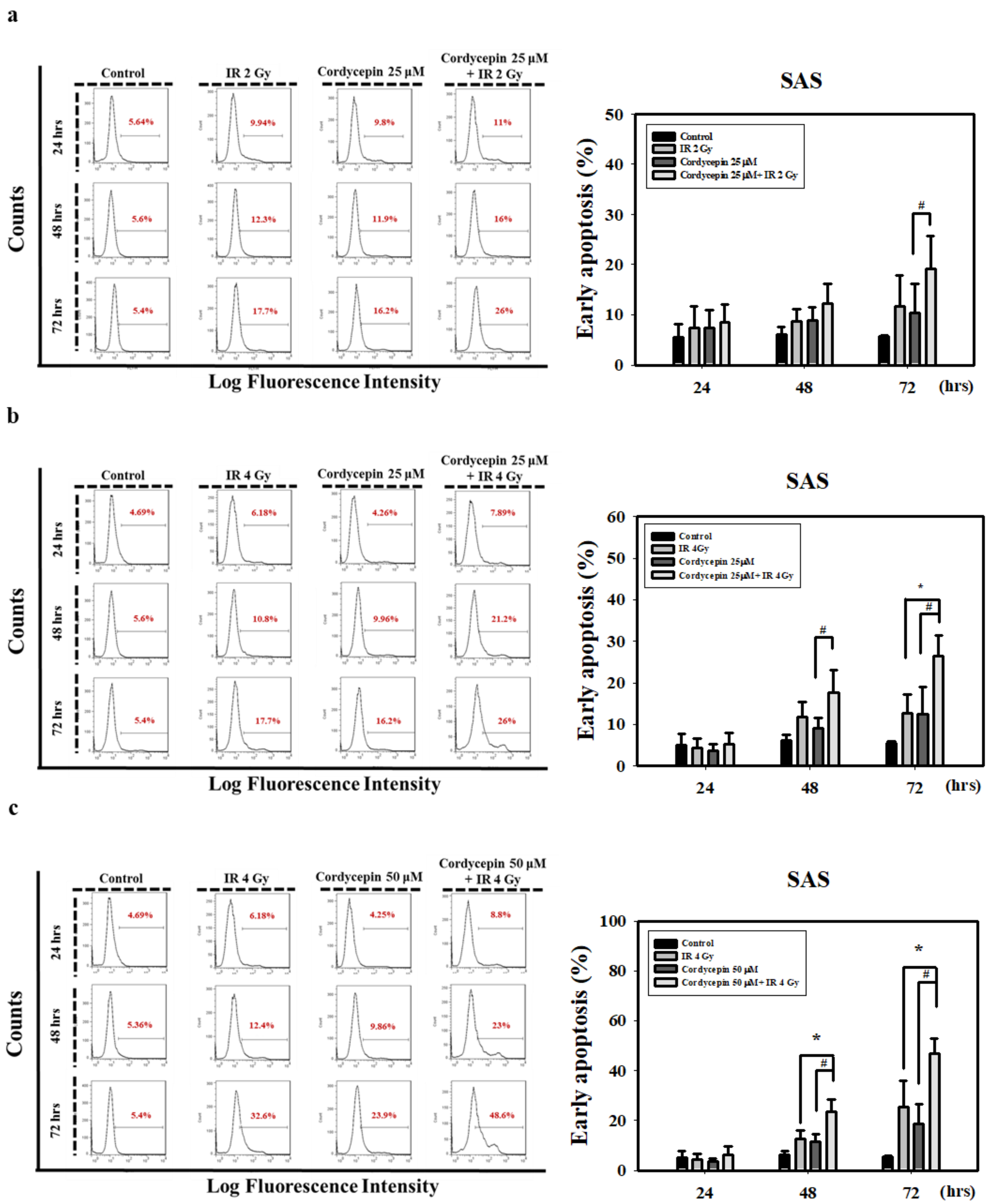

Figure S1. Measurement of apoptosis in SAS cells receiving various treatments. Early apoptosis detection was measured by flow cytometry with an annexin V apoptosis detection kit. (**a**) Cells were treated with 2 Gy IR and/or 25 μM cordycepin for 24, 48 and 72 h. (**b**) Cells were treated with 4 Gy IR and/or 25 μM cordycepin for 24, 48 and 72 h. (**c**) Cells were treated with 4 Gy IR and/or 50 μM cordycepin for 24, 48 and 72 h. $^{\#}p < 0.05$, cordycepin versus IR + cordycepin. $^{*}p < 0.05$, IR versus IR + cordycepin.

a

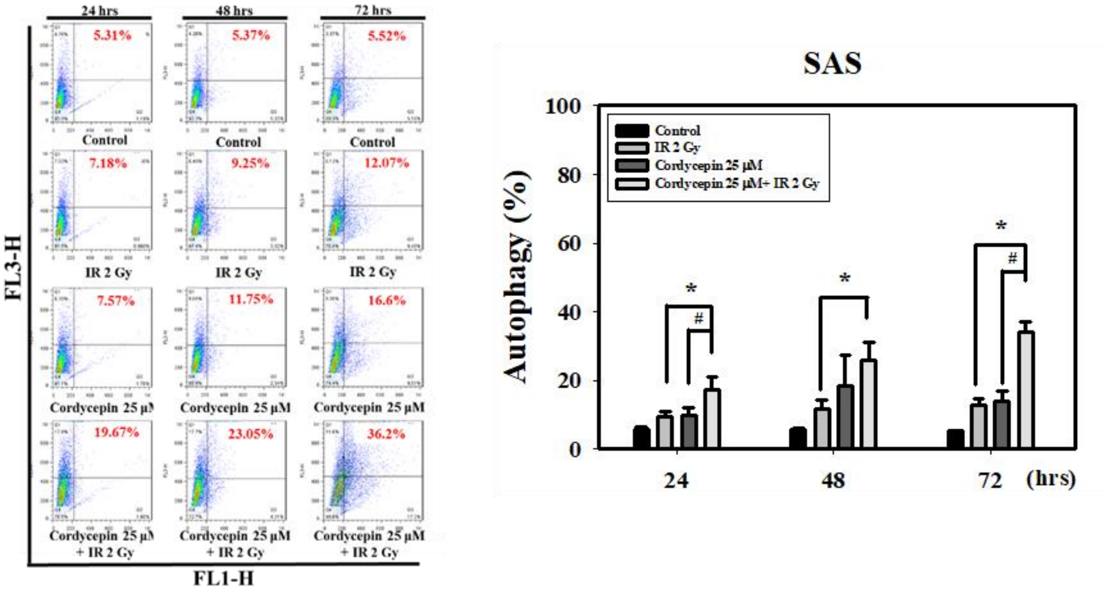

b

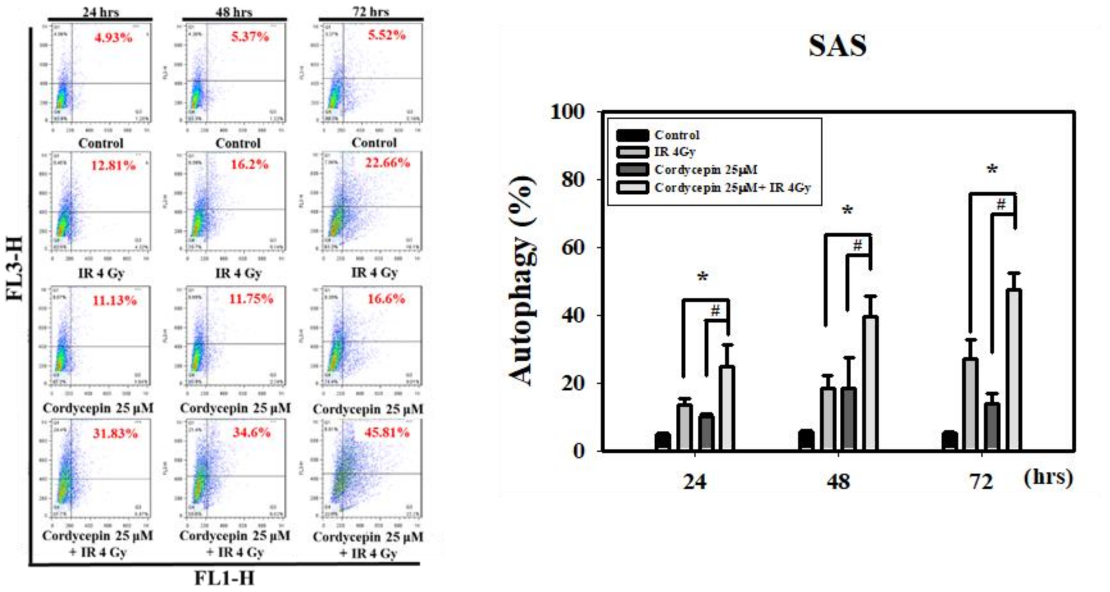

Figure S2. Measurement of autophagy in SAS cells receiving various treatments. Measurement of autophagy in SAS cells. Detection of green and red fluorescence in AO-stained cells using flow cytometry. Quantification of AVOs with AO using flow cytometry. (**a**) SAS cells were treated with IR (2 Gy) and/or cordycepin (25 μM) for 24, 48 and 72 h. (**b**) SAS cells were treated with IR (4 Gy) and/or cordycepin (25 μM) for 24, 48, and 72 h. $^{\#}p < 0.05$, cordycepin versus IR + cordycepin. $^{*}p < 0.05$, IR versus IR + cordycepin.
